# Supplementary material for: Causal Relationship between Aging and Anorexia Nervosa: A White-Matter-Microstructure-Mediated Mendelian Randomization Analysis
Source: Biomedicines. 2024 Aug 16;12(8):1874. doi: 10.3390/biomedicines12081874 (PMC11351342; doi:10.3390/biomedicines12081874)
Supplement: Supplementary file 1 [file biomedicines-12-01874-s001.zip › Supplementary Figure/Figure S8/Leaveoneout plot of MR analysis for TL to ubm-b-1658.pdf]

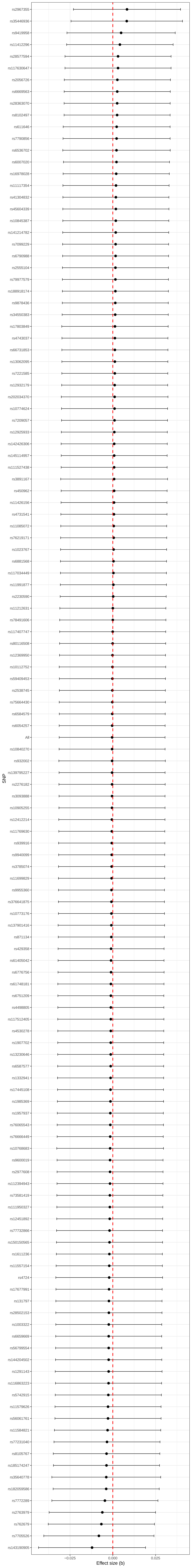
rs2967355
rs35446936
rs9419958
rs11412296
rs28577594
rs117630647
rs2056726
rs6669563
rs28363070
rs8102497
rs611646
rs7790856
rs6536702
rs6007020
rs16978028
rs11117354
rs41304832
rs45604339
rs10845387
rs114214782
rs7099229
rs6790988
rs2555104
rs79977579
rs188918174
rs9878436
rs34550383
rs17803849
rs4743037
rs66731853
rs13062095
rs7221585
rs12932179
rs202034370
rs10774624
rs7209057
rs12925933
rs142426306
rs145114957
rs111527438
rs3891167
rs450962
rs11426156
rs4731541
rs11085072
rs76219171
rs1023767
rs6881568
rs117034449
rs11991877
rs2230590
rs11212631
rs78491606
rs117407747
rs80116508
rs12369950
rs10112752
rs59409453
rs2538745
rs75664430
rs6584579
rs6054257
A8
rs10840270
rs932002
rs139795227
rs2276182
rs3093888
rs10905255
rs12412214
rs11769630
rs939916
rs9940099
rs3785074
rs11699829
rs9955360
rs376641875
rs10773176
rs137901416
rs871134
rs429358
rs61405042
rs6776756
rs61748181
rs6751209
rs4498805
rs117512405
rs4530278
rs1907702
rs13230646
rs6587577
rs1332941
rs17445108
rs1985369
rs1957937
rs76065543
rs76666449
rs10768683
rs9600019
rs2977608
rs112394943
rs73581419
rs111950327
rs12451892
rs77732866
rs150150565
rs1611236
rs11557154
rs4724
rs17677991
rs131797
rs28502153
rs1003322
rs6659669
rs56799554
rs144204502
rs1291143
rs116863223
rs5742915
rs11579626
rs56061761
rs11584821
rs77231040
rs8105767
rs185174247
rs35640778
rs182059586
rs7772289
rs2763979
rs762879
rs7705526
rs143190905
SNP
-0.025
0.000
0.025
Effect size (b)
